# The Opioid Abuse Risk Screener predicts aberrant same-day urine drug tests and 1-year controlled substance database checks: A brief report

**DOI:** 10.1177/2055102917748459

**Published:** 2017-12-22

**Authors:** Lynnette A Averill, Christopher L Averill, Lyndsay A Staley, JL Ozawa-Kirk, John SK Kauwe, Patricia Henrie-Barrus

**Affiliations:** 1 US Department of Veterans Affairs, USA; 2 Yale University, USA; 3 Brigham Young University, USA; 4 The University of Utah, USA

**Keywords:** assessment, chronic pain, controlled substance, controlled substance database, machine learning, opioid abuse, prescription drug abuse, risk stratification, scale, urine drug testing, validation

## Abstract

The Opioid Abuse Risk Screener was developed to support well-informed decision-making in opioid analgesic prescribing by extending the breadth of psychiatric risk factors evaluated relative to other non–clinician-administered measures. We examined the preliminary predictive validity of the Opioid Abuse Risk Screener relative to the widely used Screener and Opioid Assessment for Patients with Pain–Revised in predicting aberrant urine drug tests and controlled substance database checks. The Opioid Abuse Risk Screener is significantly different from the Screener and Opioid Assessment for Patients with Pain–Revised in predicting aberrant same-day urine drug tests (*Z* = 2.912, *p* = 0.0036) and controlled substance database checks within 1 year of assessment (*Z* = 3.731, *p* = 0.0002). Promising preliminary analyses using machine learning methods are also discussed.

## Introduction

Prescription opioid drug abuse continues to be a major public health concern globally (Manchikanti et al., 2017). The American Society of Interventional Pain Physicians (ASIPP) recently published guidelines for responsible, safe, and effective prescription of opioids for individuals with chronic non-cancer pain (Manchikanti et al., 2017). Consistent with previously published recommendations and proposals (Chou et al., 2009a, 2009b, 2009c; Furlan et al., 2010; Graziotti and Goucke, 1997; Jovey et al., 2003; Kalso et al., 2003; US Food and Drug Administration, 2012), these 2017 guidelines detail initial steps that should be taken to ensure safe and effective opioid therapy including, but not limited to, the following: comprehensive assessment and documentation; screening for opioid abuse to identify opioid abusers or those at risk of abuse; establishing appropriate physical and psychological diagnosis if available; considering appropriate imaging, physical diagnosis, and psychological status to collaborate with subjective complaints; and stratifying patients based on risk (Manchikanti et al., 2017).

As noted in our original report of the Opioid Abuse Risk Screener (OARS) development (Henrie-Barrus et al., 2016), there are several currently available measures designed to assess risk of misusing opioids (e.g. Butler et al., 2008, 2009; Passik et al., 2000; Webster and Webster, 2005). Although these measures provide useful information regarding potential risk of opioid abuse, they are limited in scope and do not evaluate many psychiatric variables, which seems an oversight given what the literature tells us about these risk factors for opioid abuse (Alford and Livingston, 2013; Ballantyne and Mao, 2003; Becker et al., 2008; Braden et al., 2009; Cicero et al., 2009; Edlund et al., 2007a, 2007b, 2010a, 2010b; Richardson et al., 2012; Seal et al., 2012; Sehgal et al., 2012; Sullivan et al., 2005, 2006). Furthermore, a recent report by the Centers for Disease Control and Prevention (CDC) highlights the critical importance of evaluating psychiatric variables as these both increase risk of prescription drug misuse and interfere with the resolution of pain, thus drawing out the need for continued pharmacologic intervention (Dowell et al., 2016). This has potential to create a vicious cycle in which the psychiatric variables increase risk and/or hinder pain resolution, and the ongoing pain exacerbates psychiatric symptoms, further increasing potential of abuse or other adverse events. This CDC report also noted concern regarding the accuracy and insufficiency of currently available risk assessment tools (Dowell et al., 2016) based, in part, on reports comparing risk screening methods in predicting discharge from opioid treatment with inconsistent results across and within measures (Jones et al., 2012; Moore et al., 2009).

The OARS (Henrie-Barrus et al., 2016) was developed in response to the need for a more comprehensive assessment tool that evaluates not only substance use history and aberrant behaviors, but also, importantly, psychiatric variables known to be relevant to opioid misuse and abuse (e.g. depressive and anxiety symptoms, exposure to trauma/posttraumatic stress disorder (PTSD), history of abuse/neglect, tobacco use, impulsivity, maladaptive coping, and endorsement of self-medicating behaviors). The original OARS manuscript documented preliminary yet promising evidence for effective risk stratification using a bifactor model (one general factor for opioid abuse risk comprises five domain-specific factors including anxiety, depression, traumatic stress, medical noncompliance, and substance use history) (Henrie-Barrus et al., 2016). Data collection is ongoing in support of further investigations regarding the psychometric properties of the OARS.

Given the urgent need for effective screening and risk stratification tools, the utility of additional preventative and monitoring procedures such as urine drug testing (UDT) (Christo et al., 2011; Owen et al., 2012) and controlled substance database (CSDB) checks (Manchikanti et al., 2017; Schwarz et al., 2016) and mounting need to demonstrate medical necessity for these measures for insurance reimbursement purposes (Owen et al., 2012); our primary aim was to conduct a preliminary evaluation of the predictive validity of the OARS. Specifically, we wanted to examine the ability of the OARS to predict two real-world behavioral indicators of opioid risk, aberrant UDTs, and CSDB checks. As an exploratory aim, we also compared the predictive validity of the OARS to that of the widely used Screener and Opioid Assessment for Patients with Pain–Revised (SOAPP-R) (Butler et al., 2008) in the same sample. Given its more comprehensive assessment of both emotional and behavioral factors, beyond substance use and aberrant behaviors, we hypothesized that the OARS would be significantly better than chance in predicting these real signals/outcomes. We also hypothesized that OARS would have comparable, or superior, predictive power in identifying aberrant UDT and CSDB checks compared to the SOAPP-R due to the SOAPP-R’s lack of comprehensive psychiatric and behavioral risk variables. Finally, machine learning methods are growing in popularity in medical and psychiatric research due to their ability to learn from a data set to yield more consistent, robust, and insightful results (Acion et al., 2017; Ahn and Vassileva, 2016; Kalyanam et al., 2017; Karstoft et al., 2015a, 2015b; Kessler et al., 2015; Pan et al., 2017; Youyou et al., 2015). However, these methods often require very large data sets, so our second exploratory aim was to test the feasibility of using a machine learning algorithm to evaluate psychometric properties of the OARS in a small-to-moderate sample size, similar to traditional psychiatric research populations. This study was reviewed by the University of Utah Institutional Review Board.

## Methods

### Participants

Archival data were obtained from 612 patients who completed the OARS as part of routine clinical practice. All participants were included in previous analyses (Henrie-Barrus et al., 2016). Briefly, data were collected from patients presenting to a community-based pain management clinic in the Western United States. Patients’ age ranged from 18 to 85 years with a mean age of 44.5 years. About 54 percent of the present sample self-identified as female, 45 percent male, and 0.3 percent did not identify as either male or female. Patient data were excluded if they did not complete a UDT on the same day as OARS assessment (*n* = 250). Because we also wanted to compare the predictive power of the OARS and SOAPP-R, and in order to limit any potential systematic bias between those patients who completed the OARS versus those who completed both the OARS and the SOAPP-R, those patients who completed only one risk assessment were excluded (*n* = 87). Analysis of UDT was thus conducted upon a sample of 363 patients. For CSDB analyses, we further excluded any patients for whom no CSDB check was completed within 1 year of the OARS assessment (*n* = 193) leaving a sample of 169 patients for CSDB analysis. Demographics for the UDT sample are gender (female = 52%, male = 47%, other = 0.3%) and age (19–82, mean = 42.6 years). Demographics for the CSDB sample are gender (female = 53%, male = 47%) and age (19–70, mean = 42.5 years).

### Clinical assessments

#### OARS

The OARS (Henrie-Barrus et al., 2016) is a 28-item self-report scale designed to evaluate risk of opioid misuse based on a relatively comprehensive item pool grounded in empirical evidence assessing biopsychosocial factors and aberrant behaviors. Items are rated on a 0 to 3 likert-type scale with response anchors ranging from strongly disagree to strongly agree. Possible total score ranges from 0 to 84, and a higher score indicates elevated opioid abuse risk.

#### SOAPP-R

The SOAPP-R (Butler et al., 2008) is a 24-item conceptually and empirically derived self-report scale developed to evaluate opioid risk and aberrant medication-related behaviors. Items are rated 0 to 4, using the response anchors “never,” “seldom,” “sometimes,” “often,” and “very often.” Possible total score ranges from 0 to 96, and a higher score indicates greater probability or risk of aberrant medication-related behavior.

#### UDT

Patients were asked to provide a urine sample for toxicology screening on the day of their intake visit. More specifically, they were asked to provide approximately 30–75 mL of urine in the clinic restroom, without supervision. They were asked to disclose any prescribed or non-prescribed substances that would likely appear in the testing results. We would expect some patients to have a UDT positive for opioids or other controlled substances or perhaps will have a prescription given in an emergency room or another physician. Additionally, an aberrant result could include the lack of a positive result for a substance the patient reported being prescribed and noted they are taking as this could suggest behaviors such as diversion. Since not all positive UDTs would represent a risky or aberrant behavior, we defined *aberrant UDT* to mean *any unexpected result*. An aberrant result would include anything that was inconsistent with the expectations of the clinical team at the time of testing, based on patient report, prescribed medications, and any other data available. Rating of aberrant or non-aberrant was made, with strict adherence to this definition, by the clinic staff conducting the chart reviews.

#### CSDB checks

The CSDB collects data on dispensation of schedule II–V drugs from all known outlets, including retail, institutional, and outpatient hospital pharmacies, and in- and out-of-state mail order pharmacies. Authorized prescribers and other individuals can access this information to identify potential cases of misuse, drug over-utilization, and over-prescribing. Given that many patients may take the OARS or SOAPP-R as part of their intake visit for chronic pain before receiving their first prescription for opioid analgesics, we examined CSDB checks up to 1 year following the date of the OARS, SOAPP-R, and UDT administration. The definition of aberrant versus non-aberrant was similar to that employed for UDT checks. Results were marked as aberrant if they were inconsistent with expectations of the clinical team based on available data and patient interviews or if they are generally considered to signify risk of “doctor shopping” or other illegal behaviors (number of doctors, number and timing of prescriptions, etc.). Rating of aberrant or non-aberrant was made, with strict adherence to this definition, by the clinic staff conducting the chart reviews.

#### Data analysis

Receiver operating characteristic (ROC) curve analysis was used as an index of model performance, specifically the sensitivity and specificity of the OARS and SOAPP-R in predicting aberrant UDTs and CSDB checks. ROC curve analysis is a fundamental tool for evaluating the diagnostic performance of psychometric tests and screening assessments. The area under the curve (AUC) provides a measure of the ability of the test to correctly predict the binary classification of a subject. ROC curves with either OARS or SOAPP-R as a predictor variable were conducted using the DeLong method from the pROC package in R (DeLong et al., 1988).

Support vector machines (SVMs) are a supervised machine learning algorithm that can be used to create discriminant classifications from labeled training data (i.e. providing input data such as assessment scores and classification data such as aberrant or non-aberrant UDT). We selected SVM as our exploratory machine learning method because it gave the highest accuracy for our specific data set. We used a 10-fold cross-validation approach to assess the feasibility (stability and confidence of results) of and inform future use of machine learning techniques on data sets of similar size. Finally, for each SVM, feature selection was conducted to evaluate the most stable and predictive items using sequential forward search where *α* = 0.01.

## Results

### Risk assessment predicts aberrant UDT and CSDB

**Figure 1.**
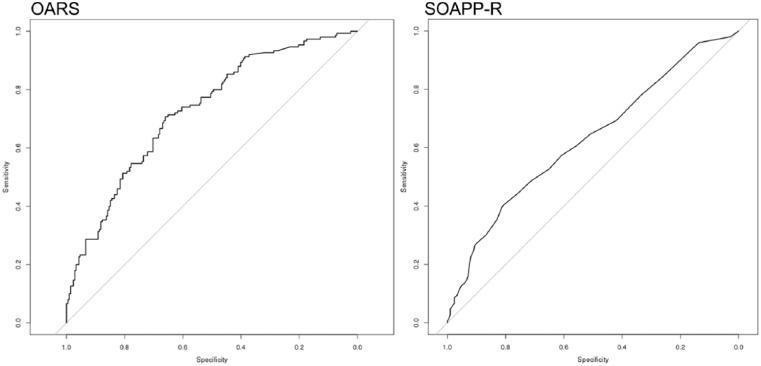
Predictive validity relative to aberrant UDTs.

**Figure 2.**
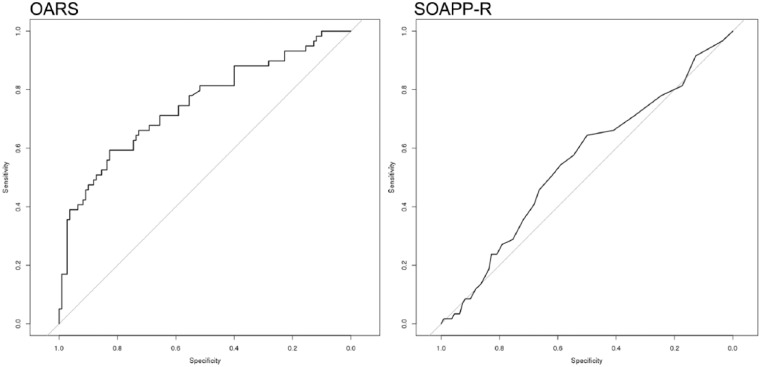
Predictive validity of identifying aberrant CSDB checks.

Results of our primary analyses indicate that the OARS (AUC = 0.727) is significantly different (*Z* = 2.912, *p* = 0.0036) than the SOAPP-R (AUC = 0.628) as a predictor of aberrant same-day UDT. Aberrant CSDB checks within 1 year of assessment date were also evaluated, again demonstrating the OARS (AUC = 0.749) to be significantly different (*Z* = 3.731, *p* = 0.0002) than the SOAPP-R (AUC = 0.552) in as a predictor (Figures 1 and 2).

### Exploring application of machine learning (SVM)

Applying an SVM algorithm to test all OARS items supports the primary results, with better than chance prediction of aberrant same-day UDT (AUC = 0.626; *n* = 363) but non-aberrant CSDB checks within 1 year of assessment date in a smaller sample size (AUC = 0.422; *n* = 169). Two items (stress load and self-medicating behaviors) were selected as being the most stable and predictive of aberrant same-day UDT (AUC = 0.636; *n* = 363), while two different items (hopelessness and traumatic nightmares) were selected as most stable and predictive of aberrant CSDB checks within 1 year of assessment date (AUC = 0.590; *n* = 169).

## Discussion

The traditional AUCs reported above are significantly greater than random predictions and provide the first evidence of the OARS’ predictive validity. The fact that the AUCs are not higher for both the OARS and the SOAPP-R is not particularly surprising, given that neither assessment was designed to capture the same information as a UDT or a CSDB check. Our results suggest, at least in this particular pain population, that the OARS is significantly more predictive than the SOAPP-R with regard to both aberrant UDT to aberrant CSDB checks. A cross-validation study of the SOAPP-R previously reported an AUC of 0.74 relative to UDTs (Butler et al., 2009). It is likely our results diverge due to differences in patient characteristics and demographics, including geographical recruitment area, score distributions, or possible differences in the analytical intent and the definition of “aberrant” used to prepare the data for analyses. Although any interpretation of preliminary data should be treated with caution, these results suggest that the OARS may have sufficient predictive power to document medical necessity for UDT in some patients and may help clinicians identify patients for whom it is particularly critical to check CSDB reports for red flags. These data also indicate that the OARS may have superior predictive validity to the SOAPP-R with regard to these measures. It is possible the additional items focused on psychiatric risk factors may contribute to this increased predictive power.

The SVM analyses, although very limited by our small sample size (by machine learning standards), were quite informative with regard to feasibility. The stability and accuracy of the results, as well as the varying level of consistency with traditional AUCs using the ROC method, suggest that a sample size of 363 is bordering upon, but not quite adequate for these particular scale validation methods, and a sample of 169 is significantly under-powered to make any trustworthy classifications. When considering the feature selection conducted for both the UDT and CSDB analyses, it is interesting to take note of the item content for the most stable and predictive items. When predicting aberrant same-day UDT, a single emotional item (high stress load) and a single behavioral item (misuse of medications to alleviate emotional distress) were selected and (even if with low confidence) were nearly as predictive on their own as the SOAPP-R using traditional ROC method. In the smaller CSDB sample, two emotional symptoms (hopelessness and traumatic nightmares) were most stable and predictive, with an AUC significantly larger than the whole scale, and again, similar to the SOAPP-R using traditional methods. While the sample size was significantly too small to take the CSDB SVM results too seriously, it is again an interesting pattern that emotional items are so strongly predictive of two gold standard real-world risk factors. These findings in particular may lend additional support to the CDCs urging for clinicians to evaluate psychological variables in a more comprehensive manner.

Informing our own future studies, and those of other investigators, our SVM experiment suggests that machine learning classification of risk may be feasible without requiring thousands of medical records to stand by the results, but that several hundred to a thousand may be required, depending on the complexity and design of the scale. Future studies of the OARS should further evaluate psychometric properties and external validity, evaluate more diverse patient populations to improve generalizability, and may benefit from using SVM or other machine learning methods to continue to optimize the assessment.

There are notable limitations for these results including the relatively small sample size (especially with regard to the machine learning analyses) and the limited diversity in the sample. The urgent need for additional methods to aid in well-informed decision-making regarding opioid analgesic prescribing practices, we felt it pertinent to provide a brief update regarding these interim results while we prepare the larger data set for additional analyses.
